# OGG1 deficiency alters the intestinal microbiome and increases intestinal inflammation in a mouse model

**DOI:** 10.1371/journal.pone.0227501

**Published:** 2020-01-14

**Authors:** Holly Simon, Vladimir Vartanian, Melissa H. Wong, Yusaku Nakabeppu, Priyanka Sharma, R. Stephen Lloyd, Harini Sampath

**Affiliations:** 1 Division of Environmental and Biomolecular Systems, Institute of Environmental Health, Oregon Health & Science University, Portland, Oregon, United States of America; 2 Oregon Institute of Occupational Health Sciences, Oregon Health & Science University, Portland, Oregon, United States of America; 3 Department of Cell, Developmental and Cancer Biology, Oregon Health & Science University, Portland, Oregon, United States of America; 4 Knight Cancer Institute, Oregon Health & Science University, Portland, Oregon, United States of America; 5 Division of Neurofunctional Genomics, Department of Immunobiology and Neuroscience, Medical Institute of Bioregulation, Fukuoka, Kyushu, Japan; 6 Department of Nutritional Sciences, Rutgers, the State University of New Jersey, New Brunswick, New Jersey, United States of America; 7 New Jersey Institute for Food, Nutrition, and Health, Rutgers, the State University of New Jersey, New Brunswick, New Jersey, United States of America; 8 Department of Molecular and Medical Genetics, Oregon Health & Science University, Portland, Oregon, United States of America; University of Illinois, UNITED STATES

## Abstract

OGG1-deficient (*Ogg1*^*-/-*^) animals display increased propensity to age-induced and diet-induced metabolic diseases, including insulin resistance and fatty liver. Since the intestinal microbiome is increasingly understood to play a role in modulating host metabolic responses, we examined gut microbial composition in *Ogg1*^*-/-*^ mice subjected to different nutritional challenges. Interestingly, *Ogg1*^*-/-*^ mice had a markedly altered intestinal microbiome under both control-fed and hypercaloric diet conditions. Several microbial species that were increased in *Ogg1*^*-/-*^ animals were associated with increased energy harvest, consistent with their propensity to high-fat diet induced weight gain. In addition, several pro-inflammatory microbes were increased in *Ogg1*^*-/-*^ mice. Consistent with this observation, *Ogg1*^*-/-*^ mice were significantly more sensitive to intestinal inflammation induced by acute exposure to dextran sulfate sodium. Taken together, these data indicate that in addition to their proclivity to obesity and metabolic disease, *Ogg1*^*-/-*^ mice are prone to colonic inflammation. Further, these data point to alterations in the intestinal microbiome as potential mediators of the metabolic and intestinal inflammatory response in *Ogg1*^*-/-*^ mice.

## Introduction

Oxidative stress can result from endogenous and exogenous generation of reactive oxygen species (ROS) in response to environmental and dietary factors. Induction of oxidative stress has been implicated in the onset and progression of a number of pathologies, including metabolic syndrome and chronic inflammation. ROS exert their effects by altering the redox status of the cell and by reacting with and damaging cellular constituents. One of the important targets of ROS-induced damage is DNA, which is subject to oxidative lesions that must be repaired to maintain genomic stability [1–3]. Oxidatively induced DNA lesions are repaired primarily by the base excision repair (BER) pathway, in which excision of the damaged bases is initiated by DNA glycosylases. The enzyme 8-oxoguanine DNA glycosylase (OGG1) removes the most prevalent DNA lesions, 7,8-dihydro-8-oxoguanine (8-oxoG) and 2,6-diamino-4-hydroxy-5-formamidopyrimidine (FapyGua) from both genomic and mitochondrial DNA [1–9]. Deficiencies in OGG1 have been associated with several diseases including cancers [10–14], neurodegenerative diseases [15–23], and type 2 diabetes [24, 25]. Our laboratory has shown that OGG1 deficiency renders mice susceptible to metabolic pathologies including obesity, insulin resistance, and ectopic lipid accumulation [26–28]. Conversely, we have shown that overexpression of a mitochondrially-targeted OGG1 results in significant protection from diet-induced obesity, indicating an important role for OGG1 activity in regulating cellular metabolic homeostasis.

The gastrointestinal tract is colonized by a large number of microorganisms, including bacteria, viruses, archaea, fungi, and protozoa. These microorganisms are collectively referred to as the gut microbiome and have now been demonstrated to serve a variety of functions, including energy harvest, xenobiotic metabolism, vitamin production, and immune function. Accordingly, aberrant intestinal microbial colonization, or intestinal dysbiosis, has been implicated in numerous pathologies, including the development of obesity [29–36]. Furthermore, the colonic environment is also subject to oxidative stress, and dysbiotic microbiota may result in further increases in amounts of reactive oxygen and nitrogen species that can induce further DNA damage [37]. While numerous studies have established that diet is a key and rapid modulator of the intestinal microbiome [38–40], it is increasingly appreciated that host genetics can also influence the gut microbial ecology as well as vulnerability to alterations in the microbiome. Furthermore, host genetic makeup can interact with diet to induce specific changes in the intestinal microbiota that alter disease risk [41].

Given our prior observations of increased propensity to obesity in OGG1-deficient mice, we sought to determine if OGG1 status, in the context of a regular low-fat diet or a hypercaloric diet, impacts intestinal microbial composition and whether any observed changes are associated with disease risk.

## Methods

### Animals and sample collection/DNA extraction

The generation of *Ogg1*^*-/-*^ mice has been previously described [26]. WT and *Ogg1*^*-/-*^ mice on a C57Bl6 background were used for these studies. This study was carried out in strict accordance with the recommendations in the Guide for the Care and Use of Laboratory Animals of the National Institutes of Health. The protocol was approved by the Institutional Animal Care and Use Committee of Oregon Health & Science University. For this study, six male wild-type (WT) and *Ogg1*^*-/-*^ mice on a C57Bl6 background were weaned onto a standard chow diet (Picolab Laboratory Rodent Diet (5L0D), Purina Mills). Starting at 12 weeks of age, animals were individually housed and were either continued on the chow or randomized to a high fat diet (HFD) regimen. The HFD (Research Diets 12492) derived 60% of its calories from fat. Fresh fecal samples were collected after 5 weeks of feeding in the individually housed setting, when mice were 20 weeks of age. Fecal samples were frozen at -80°C before shipping on dry ice to Molecular Research (Lubbock, TX) for DNA extraction. Total DNA was extracted and purified from murine feces at Molecular Research (Lubbock, TX), using the Mo-Bio Power Soil kit (Qiagen, Valencia, CA) according to the manufacturer's protocol.

#### 16S rRNA gene amplicon sequencing and sequence processing

Barcoded amplicon sequencing (bacterial tag encoded FLX-Titanium amplicon pyrosequencing (bTEFAP)) was performed as described [42]. In brief, the purified DNA was used as starting material for a single-step 30 cycle PCR using HotStarTaq Plus Master Mix Kit (Qiagen, Valencia, CA) and barcoded 16S primers (variable region V1-V3 using primer pair 27F-519R) under the following cycling conditions: cycle1: 94°C for 3 minutes; cycles 2–29: 94°C for 30 seconds; 53°C for 40 seconds and 72°C for 1 minute; cycle 30, followed by a final elongation step at 72°C for 5 minutes. Next, PCR amplicon products from different samples were mixed in equal concentrations and purified using Agencourt Ampure beads (Agencourt Bioscience Corporation, MA, USA). Finally, sample pools were sequenced using Roche 454 FLX titanium instruments and reagents following the manufacturer’s guidelines. Sequence processing included removal of sequences < 200 bp, sequences with ambiguous base calls, and sequences with homopolymer runs > 6 bp. Sequences were denoised [43] and Operational Taxonomic Units (OTUs) were defined via a clustering algorithm at 3% divergence (97% similarity) [44]. To compare samples with different sequencing depths, each sample was rarefied to the same number of reads (1460 sequences).

### Taxonomic assignment and statistical analysis

Taxonomy was assigned in MACQIIME using the Greengenes taxonomy and a Greengenes reference database (currently version 12_10) [45], following the default taxonomy assignment method with the RDP Classifier 2.2 [46]. Statistical analyses were performed in Excel (Version 14.7.3) or in R [47]. Tests of significance in mean comparisons were performed using two-sided Student's two-sample t-tests or Analysis of Variance (ANOVA) and Tukey's posthoc test. The nonparametric *p*-values were calculated using 999 Monte Carlo permutations. The Bonferroni correction for multiple comparisons [48] was applied as needed.

### Alpha and beta diversity analysis

Alpha and beta diversity measures were calculated using the Quantitative Insights Into Microbial Ecology (QIIME) pipeline (QIIME 1.8.0) [49]. Diversity analyses included Chao1 [48], Goods Coverage [50], observed species, Faith's Phylogenetic Diversity (Faith [51], the Shannon (H´) [52] and Simpson Diversity Indices [53]. Bray–Curtis dissimilarity matrixes [54] were generated from normalized data and Principle Co-ordinate Analyses (PCoA) [55] were performed using MACQIIME with default settings. As described in [56], UniFrac was also used to assess differences among samples, using a distance matrix to cluster the samples with UPGMA [57] and to perform PCoA [58].

### Dextran sodium sulfate-induced colitis

Dextran sodium sulfate (DSS) was purchased from TdB Consultancy AB, Uppsala, Sweden and dissolved at a final concentration of 3% (w/v) in sterile water containing 5% (w/v) dextrose. Male WT and *Ogg1*^*-/-*^ mice (24 weeks) were weighed and group housed with a maximum of 5 mice per cage. Chow diet was available *ad libitum*, and mice were randomized to receive either 5% dextrose-supplemented water or DSS-containing water. The acute DSS challenge was continued for 5 days during which body weights, liquid consumption, and overall health were assessed daily. After 5 days on DSS-supplemented water, all mice were euthanized by CO_2_, followed by removal of the colon. Tissues were washed in 1x PBS and longitudinal incisions were made to open the intestinal tube and facilitate pinning the tissue lumen side up onto wax plates for photographic documentation and further processing for histopathologic analyses. Full length sections of each colon were formalin-fixed, embedded, and processed for H&E staining. All slides were scored blindly by two independent investigators, with individual scores given for goblet cell depletion, mucosal thickening, and inflammatory infiltration.

#### Measurement of DNA base lesions

DNA base lesions in isolated DNA samples were measured using gas chromatography-tandem mass spectrometry (GC-MS/MS) with isotope-dilution as described previously [59].

#### Serum cytokine analysis

Plasma was collected from each animal before DSS treatment and after 5 days of treatment. A mouse-specific cytokine kit (10-plex, LuminexCorp.) was used according to the manufacturer’s recommendations on a Luminex 200 analyzer. Quality control samples, standards, and plasma were run in duplicate on the same plate. Plates were analyzed using a dedicated plate reader and xPONENT software. Cytokines that were within limits of detection are reported.

## Results

### Increased body weight and adiposity in *Ogg1*^*-/-*^ mice fed a HFD

**Fig 1:**
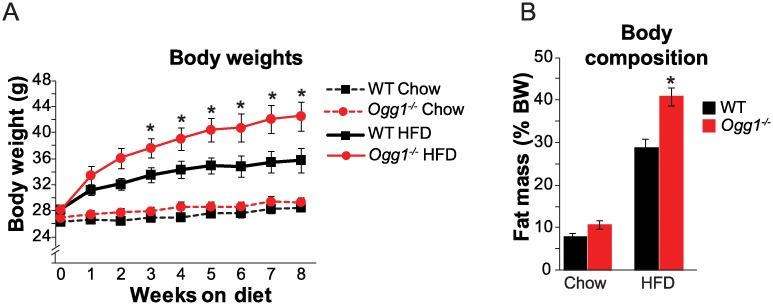
Body weight and composition on chow and HFD. WT and *Ogg1*^*-/-*^ 12-week old male mice were given ad libitum access to chow or HFD for 8 weeks. (A) Mice were weighed weekly during and (B) body composition was measured by NMR at the end of 8 weeks of feeding. n = 6 animals per cohort. *, p<0.05 vs. WT.

We have previously reported that HFD-fed *Ogg1*^*-/-*^ mice are prone to diet-induced obesity and adiposity, relative to WT controls [26, 27]. In the current study, we were interested in determining if differences in adiposity would be correlated with alterations in the microbiome that are known to predispose animals to metabolic pathologies. Therefore, we first established a model of obesity by *ad libitum* administration of a high-fat diet (HFD) deriving 60% of its calories from fat. Control animals were fed a standard low-fat chow diet. Starting at 12 weeks of age, WT and *Ogg1*^*-/-*^ mice were individually-housed and fed chow or HFD for 8 weeks. Body weights were measured weekly, and body composition was measured after 8 weeks of feeding. Fecal samples were collected after 6 weeks of feeding. As with previous cohorts [26, 60], *Ogg1*^*-/-*^ mice gained more weight (Fig 1A) and more fat mass (Fig 1B) upon HFD-administration, relative to WT controls.

### Microbiota analysis

After 6 weeks of feeding, mice were transferred to a clean cage, and fecal samples were collected and flash frozen to investigate changes in the gut microbiota. In collaboration with the Mouse Metabolic Phenotyping Center (MMPC) at UC Davis, the V1-V3 variable region of the 16S rRNA gene was examined by bacterial tag encoded FLX-Titanium amplicon pyrosequencing (bTEFAP) as described [61]. A mean of 5450.5 ± 280 (SEM) individual sequencing reads was obtained per sample. After quality control, including removal of singleton sequences and chimeras [62–65], an average number of 3560 ± 256 (SEM) sequences for each sample were passed through to OTU classification. Final OTUs were taxonomically classified using BLASTn [66] against a proprietary curated database derived from GreenGenes (Caporaso [67] [68], RDPII [69] and NCBI [38]. Alpha and beta diversity measures were calculated using the QIIME pipeline (QIIME 1.8.0) [49]. The rarefaction curves and sequencing coverage (Goods coverage index > 0.99 across samples [50]) (S1 Fig) indicated that adequate sequencing depth was achieved.

### Taxonomic profiles

**Fig 2:**
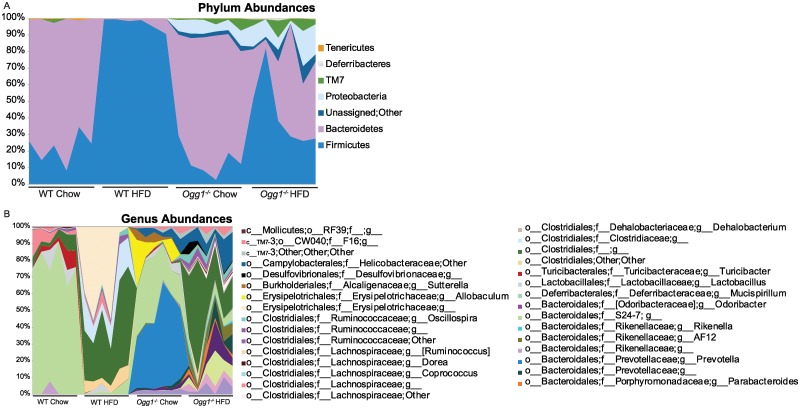
Diversity area graphs. Data show diversity of the gut microbiota recovered from chow and HFD-fed WT and *Ogg1*^*-/-*^ mice at the (A) phylum and (B) genus levels. Results are from the analysis of microbial sequences recovered from the fecal samples as described in Methods. Taxonomy was assigned in MACQIIME using the Greengenes taxonomy and a Greengenes reference database (currently version 12_10) [45], following the default taxonomy assignment method with the RDP Classifier 2.2 [46].

Taxonomic designations were defined as query sequences that shared identity with reference sequences within the range of (i) 77–80% (phylum); (ii) 80–85% (class); (iii) 85–90% (order); (iv) 90–95% (family); (v) 95–97% (genus); and (vi) >97% (species). Area graphs indicating diversity at the phylum and genus levels are shown in Fig 2A and 2B, respectively. In these results, a significant increase in the taxonomic diversity of gut microbiota is apparent in both chow-fed and HFD-fed *Ogg1*^*-/-*^ mice, relative to WT controls. As predicted from prior reports, HFD-feeding reduced diversity in both genotypes, but *Ogg1*^*-/-*^ mice retained a significantly greater microbial diversity even after HFD-feeding, relative to WT controls. Two-way ANOVA with *ad hoc* Tukey's test was performed for significance and results are shown in Tables 1 and 2 and described below.

**Table 1:** Composition of gut microbiota at the phylum level.

Phylum level	WT		*Ogg1*^*-/-*^		*p*-value		
	Chow	HFD	Chow	HFD	Genotype	Diet	G×D^1^
Firmicutes	21.9 ± 3.77^b^^c^	97 ± 1.45^a^	13.8 ± 3.79^c^	42.4 ± 8.87^b^	<0.001	<0.001	<0.001
Bacteroidetes	77.4 ± 3.92^a^	3 ± 1.45^c^	74.2 ± 3.78^a^	36.8 ± 8.42^b^	0.007	<0.001	0.002
Proteobacteria	0 ± 0^b^	0 ± 0^b^	7.26 ± 0.748^a^	11.9 ± 2.9^a^	<0.001	0.134	0.134
TM7	0.398 ± 0.398^a^^b^	0 ± 0^b^	2.28 ± 1.1^a^^b^	4.25 ± 1.6^a^	0.006	0.435	0.245
Unassigned;Other	0 ± 0^b^	0 ± 0^b^	2.46 ± 0.174^a^^b^	4.12 ± 1.59^a^	0.001	0.313	0.313
Deferribacteres	0 ± 0^b^	0 ± 0^b^	0.015 ± 0.00974^b^	0.468 ± 0.189^a^	0.019	0.027	0.027
Tenericutes	0.35 ± 0.141^a^	0 ± 0^b^	0 ± 0^b^	0 ± 0^b^	0.022	0.022	0.022

Values are mean percentages ± SEM, n = 6 per treatment group.

^a-c^Mean percentages in rows without a common superscript letter differ (P < 0.05) as analyzed by two-way ANOVA and the TUKEY test.

^1^G × D = Genotype × Diet interaction effect.

**Table 2:** Taxon-level composition of gut microbiota.

Phylum/Family/Genus	WT		*Ogg1*^*-/-*^		*p*-value		
	Chow	HFD	Chow	HFD	Genotype	Diet	G×D^1^
Firmicutes_Clostridia_ Clostridiales	5.76 ± 1.36^b^	37.8 ± 6.36^a^	0.601 ± 0.2^b^	30.6 ± 8.04^a^	0.243	<0.001	0.84
Firmicutes_Clostridia_ Clostridiales_Clostridiaceae	0.0845 ± 0.0771^b^	14.3 ± 2.57^a^	0.125 ± 0.0737^b^	0.18 ± 0.0526^b^	<0.001	<0.001	<0.001
Firmicutes__Clostridia__Clostridiales_Other	0 ± 0^b^	6.38 ± 1.06^a^	0 ± 0^b^	1.09 ± 0.824^b^	0.001	<0.001	0.001
Firmicutes_Clostridia_ Clostridiales_ Ruminococcaceae	0.62 ± 0.223^a^^b^	1.67 ± 0.61^a^^b^	0.134 ± 0.134^b^	2.1 ± 0.6^a^	0.957	0.003	0.314
Firmicutes__Clostridia__Clostridiales__ Ruminococcaceae__ Oscillospira	0.309 ± 0.0511^b^	1.83 ± 0.492^a^^b^	0.506 ± 0.336^b^	3.52 ± 0.618^a^	0.039	<0.001	0.096
Firmicutes_Clostridia_ Clostridiales_ Ruminococcaceae_Other	0.0436 ± 0.0227^b^	0.218 ± 0.038^b^	0 ± 0^b^	0.794 ± 0.216^a^	0.026	<0.001	0.011
Firmicutes__Clostridia__Clostridiales__ Dehalobacteriaceae__ Dehalobacterium	0.0497 ± 0.0346^b^	0.0167 ± 0.00908^b^	0.0125 ± 0.0125^b^	0.578 ± 0.165^a^	0.006	0.005	0.002
Firmicutes__Clostridia__Clostridiales__ Lachnospiraceae_Other	0 ± 0^b^	0 ± 0^b^	0.0261 ± 0.0123^b^	1.7 ± 0.573^a^	0.007	0.008	0.008
Firmicutes__Clostridia__Clostridiales__ Lachnospiraceae__ Dorea	0 ± 0^b^	0.00673 ± 0.00455^b^	0.00981 ± 0.00981^b^	0.49 ± 0.0883^a^	<0.001	<0.001	<0.001
Firmicutes__Clostridia__Clostridiales__ Lachnospiraceae__ [Ruminococcus]	0.0256 ± 0.0121^b^	0.0238 ± 0.0183^b^	0 ± 0^b^	0.624 ± 0.15^a^	0.001	0.001	0.001
Firmicutes__Clostridia__Clostridiales__ Lachnospiraceae__ Coprococcus	0 ± 0^b^	0.127 ± 0.0168^a^	0 ± 0^b^	0.122 ± 0.0435^a^	0.911	<0.001	0.911
Firmicutes__Clostridia__Clostridiales__ Lachnospiraceae	5.67 ± 2.45^a^	2.11 ± 1.09^a^^b^	0.0963 ± 0.0286^b^	0.356 ± 0.0992^b^	0.013	0.232	0.169
Firmicutes__Bacilli__Turicibacterales__ Turicibacteraceae__ Turicibacter	4.85 ± 1.88^a^	0.116 ± 0.0561^b^	0.282 ± 0.213^b^	0 ± 0^b^	0.023	0.016	0.029
Firmicutes__Erysipelotrichi__Erysipelotrichales__Erysipelotrichaceae	0.338 ± 0.0798^b^	31.1 ± 9.91^a^	0 ± 0^b^	0 ± 0^b^	0.005	0.006	0.006
Firmicutes__Erysipelotrichi__Erysipelotrichales__Erysipelotrichaceae_ Allobaculum	0 ± 0^b^	0 ± 0^b^	9.06 ± 4.4^a^	0 ± 0^b^	0.053	0.053	0.053
Firmicutes_Bacilli_Lactobacillales_ Lactobacillaceae_Lactobacillus	4.14 ± 2.29	1.25 ± 0.848	2.91 ± 1.11	0.305 ± 0.119	0.429	0.054	0.917
Bacteroidetes__Bacteroidia__Bacteroidales__ S24-7	76 ± 4.32^a^	2.99 ± 1.45^c^	26.4 ± 4.23^b^	4.02 ± 1.06^c^	<0.001	<0.001	<0.001
Bacteroidetes__Bacteroidia__Bacteroidales__Prevotellaceae__Prevotella	0 ± 0^b^	0 ± 0^b^	44.2 ± 4.83^a^	0 ± 0^b^	<0.001	<0.001	<0.001
Bacteroidetes__Bacteroidia__Bacteroidales__ Prevotellaceae	0 ± 0	0 ± 0	1.05 ± 0.434	4.57 ± 2.35	0.029	0.156	0.156
Bacteroidetes__Bacteroidia__Bacteroidales__ Rikenellaceae	1.38 ± 1.31	0 ± 0	0.411 ± 0.199	0.386 ± 0.148	0.668	0.306	0.323
Bacteroidetes__Bacteroidia__Bacteroidales__Rikenellaceae__AF12	0 ± 0^b^	0.00513 ± 0.00513^b^	0.455 ± 0.141^b^	5.64 ± 1.15^a^	<0.001	<0.001	<0.001
Bacteroidetes__Bacteroidia__Bacteroidales__Rikenellaceae__Rikenella	0 ± 0^b^	0 ± 0^b^	0.14 ± 0.0492^b^	1.37 ± 0.416^a^	0.002	0.008	0.008
Bacteroidetes__Bacteroidia__Bacteroidales_ Other	0 ± 0^b^	0 ± 0^b^	0.0821 ± 0.0293^b^	4.02 ± 0.77^a^	<0.001	<0.001	<0.001
Bacteroidetes__Bacteroidia__Bacteroidales	0 ± 0^b^	0 ± 0^b^	0.133 ± 0.0705^b^	7.72 ± 2.98^a^	0.016	0.019	0.019
Bacteroidetes__Bacteroidia__Bacteroidales__Porphyromonadaceae__ Parabacteroides	0 ± 0^b^	0 ± 0^b^	0.0614 ± 0.0282^b^	2.43 ± 0.92^a^	0.014	0.018	0.018
Bacteroidetes__Bacteroidia__Bacteroidales__[Odoribacteraceae]__ Odoribacter	0 ± 0^b^	0 ± 0^b^	0.0701 ± 0.0341^b^	0.283 ± 0.086^a^	0.001	0.032	0.032
Bacteroidetes__Bacteroidia__Bacteroidales__Bacteroidaceae__ Bacteroides	0 ± 0^b^	0 ± 0^b^	1.25 ± 0.393^a^^b^	6.35 ± 2.63^a^	0.01	0.07	0.07
Proteobacteria__Betaproteobacteria__Burkholderiales__Alcaligenaceae__Sutterella	0 ± 0^b^	0 ± 0^b^	3.07 ± 1.05^a^	0.169 ± 0.0687^b^	0.006	0.012	0.012
Proteobacteria__Deltaproteobacteria__Desulfovibrionales__Desulfovibrionaceae	0 ± 0	0 ± 0	0 ± 0	1.9 ± 1.07	0.09	0.09	0.09
Proteobacteria__Epsilonproteobacteria__Campylobacterales__Helicobacteraceae_Other	0 ± 0^b^	0 ± 0^b^	4.19 ± 1.22^a^^b^	9.88 ± 2.99^a^	<0.001	0.093	0.093
TM7__TM7-3__CW040__F16	0.398 ± 0.398	0 ± 0	1.33 ± 0.605	1.95 ± 0.92	0.023	0.852	0.395
TM7__TM7-3_Other	0 ± 0^b^	0 ± 0^b^	0.944 ± 0.5^a^^b^	2.3 ± 0.852^a^	0.004	0.185	0.185
Unassigned_Other	0 ± 0^b^	0 ± 0^b^	2.46 ± 0.174^a^^b^	4.12 ± 1.59^a^	0.001	0.313	0.313
Deferribacteres__Deferribacteres__ Deferribacterales__ Deferribacteraceae__Mucispirillum	0 ± 0^b^	0 ± 0^b^	0.015 ± 0.00974^b^	0.468 ± 0.189^a^	0.019	0.027	0.027
Tenericutes__Mollicutes__RF39	0.35 ± 0.141^a^	0 ± 0^b^	0 ± 0^b^	0 ± 0^b^	0.022	0.022	0.022

Values are mean percentages ± SEM, n = 6 per treatment group.

^a-c^Mean percentages in rows without a common superscript letter differ (P < 0.05) as analyzed by two-way ANOVA and the TUKEY test.

^1^G × D = Genotype × Diet interaction effect.

### Phylum level profiles

In WT chow-fed mice, only two phyla, the Firmicutes and Bacteroides, were present at abundances > 1% (Table 1, Fig 2). TM7 and Tenericutes were present at lower abundances in chow-fed WT mice. After HFD-feeding, only Firmicutes and Bacteroides were detectable in WT mice, and the proportions were shifted relative to chow-fed controls. Overall, the Firmicutes increased significantly while Bacteroidetes and Tenericutes decreased significantly. Compared to WT chow-diet, the *Ogg1*^*-/-*^ chow-fed mice that were bred and reared under identical conditions showed significantly higher relative abundance of Proteobacteria and significantly lower relative abundance of Tenericutes. Gut microbiomes from *Ogg1*^*-/-*^ HFD-fed animals revealed significant increases in relative abundance of Firmicutes and Deferribacteres and a significant decrease in Bacteroidetes, compared to *Ogg1*^*-/-*^ on chow diet. Compared to WT HFD-fed mice, gut microbiomes of the *Ogg1*^*-/-*^ HFD-fed mice showed a significant decrease in relevant abundance of Firmicutes and Tenericutes and significant increases in Bacteroidetes, Proteobacteria, TM7, an unassigned group, and Deferribacteres (Table 1, Fig 2).

Results are from the analysis of microbial sequences recovered from the fecal samples of chow- and HFD-fed WT and *Ogg1*^*-/-*^ mice. Taxonomy was assigned in MACQIIME using the Greengenes taxonomy and a Greengenes reference database (currently version 12_10) [45], following the default taxonomy assignment method with the RDP Classifier 2.2 [46]. Means without a common superscript letter are significantly different (p < 0.05), as analyzed by two-way ANOVA and the TUKEY test.

### Profiles at finer taxonomic scale: *Effects of HFD-feeding in WT mice*

In chow-fed WT mice, gut microbial taxa present at ≥ 1% relative abundance revealed sequences in the Bacteroides group and were identified mainly as belonging to the Bacteroidia class, predominantly to group S24-7, with a minor amount identified as members of the family Rikenellaceae (Table 2). Several Firmicutes taxa were abundant at relatively equitable levels; this included members of the family Lachnospiraceae and an unidentified genus in the Clostridia class, and *Turicibacter* and *Lactobacillus* in the Bacilli class. In comparison, in HFD-fed WT mice, Bacteroidetes S24-7 decreased to 2.99% ± 1.45% in relative abundance, with no other Bacteroidetes taxa detected at ≥ 0.01%. Distribution of Firmicutes taxa also changed dramatically, consistent with previous demonstrations of changes in Bacteroidetes and Firmicutes upon HFD administration [70]. Significant increases were observed in the relative abundance of unidentified genera in the Clostridia and Erysipelotrichi classes. A smaller, but still significant increase was observed in the relative abundance of a second unidentified Clostridia genus, as well as decreases in abundance for genera of the Bacilli class, *i*.*e*., *Lactobacillus* and *Turicibacter* (Table 2).

Results are from the analysis of microbial sequences recovered from the fecal samples of chow- and HFD-fed WT and *Ogg1*^*-/-*^ mice. Taxonomy was assigned in MACQIIME using the Greengenes taxonomy and a Greengenes reference database (currently version 12_10) [45], following the default taxonomy assignment method with the RDP Classifier 2.2 [46]. Means without a common superscript letter are significantly different (p < 0.05), as analyzed by two-way ANOVA and the TUKEY test.

### Profiles at finer taxonomic scale: Effect of OGG1 genotype on chow diet

Major differences in the gut microbiomes of *Ogg1*^*-/-*^ compared to WT mice on chow diets included a significant increase in the relative abundance of *Prevotella* sequences (Bacteriodetes phylum) and a decrease in relative abundance of the Bacteroidetes family S24-7 in *Ogg1*^*-/-*^ animals. Significant changes in Firmicutes included an increase in relative abundance of the genus *Allobaculum* and decreases in abundance of sequences belonging to the family Lachnospiraceae and the genus *Turicibacter*. Proteobacteria abundance increased in the genus *Sutterella* and the family Helicobacteraceae, although the latter was not statistically significant (Table 2).

#### Profiles at finer taxonomic scale: Effects of HFD-feeding in *Ogg1*^*-/-*^ mice

Similar to the case for WT animals, *Ogg1*^*-/-*^ HFD gut microbiomes showed a decrease in Bacteroidetes S24-7 taxa compared to chow-fed counterparts. However, HFD-fed *Ogg1*^*-/-*^ mice showed significant increases in the relative abundance of some of the other Bacteroidetes taxa, including the genera AF12, *Parabacteroides*, *Rikenella* and two unidentified genera in the Bacteriodia class (Table 2). Significant changes in Firmicutes in HFD-fed *Ogg1*^*-/-*^ mice included increases in relative abundance of several taxa in the Clostridia class, one unidentified genus, as well as the genus *Oscillospira*, and taxa from the families Ruminococcaceae and Lachnospiraceae. Significant decreases in relative abundance were observed for Firmicutes genera *Lactobacillus* and *Allobaculum*, members of the Bacilli and Erysipelotrichi classes, respectively. A Betaproteobacterial class member belonging to the genus *Sutterella*, seen only in *Ogg1*^*-/-*^ animals, was significantly decreased by HFD-feeding (Table 2).

#### Effects of host genotype and diet

At the phylum level, changes in the abundance of all major bacterial groups detected showed a significant host genotype effect (*p* < 0.05, Table 1). Furthermore, there was a significant diet effect noted for the abundance of Firmicutes, Bacteroidetes, Deferribacteres and the Tenericutes. A significant genotype × diet interaction was also observed for these groups (Table 1). At family and genus levels, there were significant effects of host genotype on the abundance of a variety of taxa across all phyla present. Some of the more notable changes were observed for S24-7 and *Prevotella* (Bacteroidetes phylum), as well as taxa in families Helicobacteraceae (Proteobacteria phylum), and the orders Clostridia and Erysipelotri (both Firmicutes phylum). Significant effects of diet were also observed, with the taxa most dramatically affected identified as S24-7 and *Prevotella* (Bacteroidetes phylum), and members of the Clostridia class (Firmicutes phylum). All of these taxa showed significant effects for the interaction of genotype × diet, although the Prevotella and the Clostridia were not consistent across all identified members (Table 2).

#### Within sample (alpha) diversity

**Fig 3:**
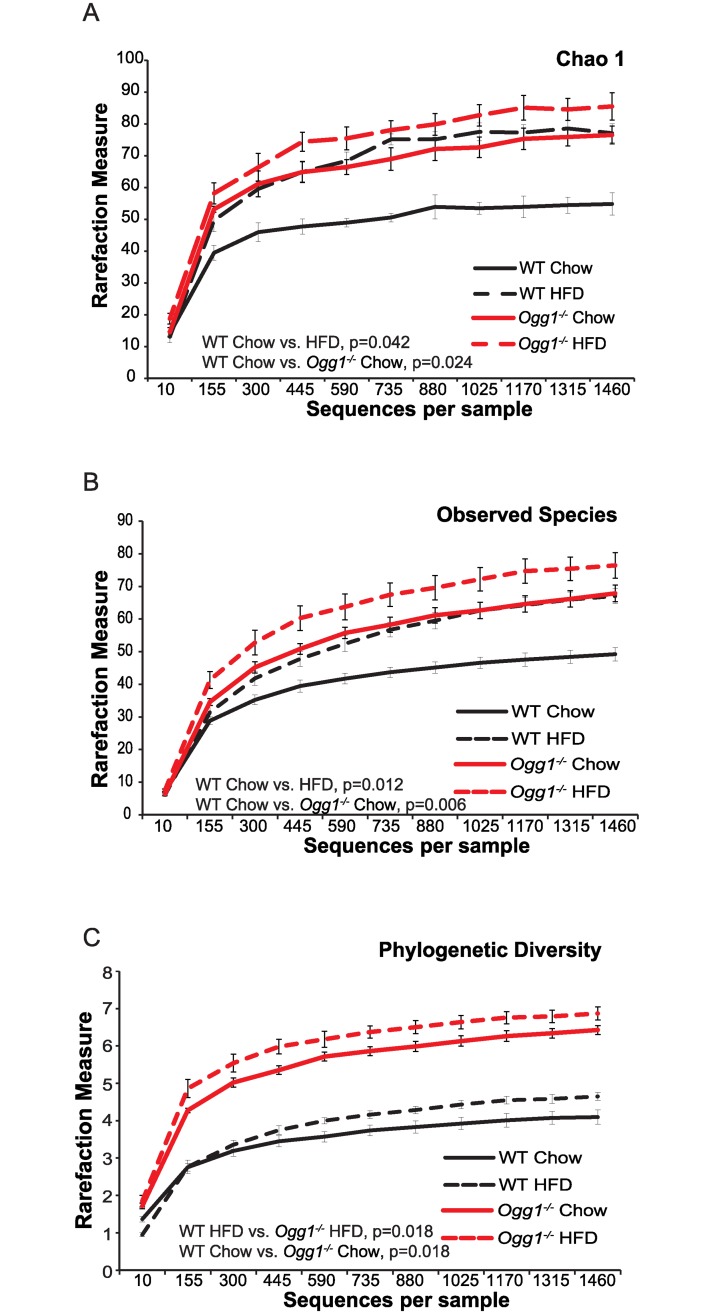
Alpha diversity measures of gut microbiota from chow- and HFD-fed WT and *Ogg1*^*-/-*^ mice. Alpha diversity measures are shown for WT and *Ogg1*^*-/-*^ animals. Results are from the analysis of microbial sequences recovered from the fecal samples as described in Methods. Significant differences were observed using Bonferroni-corrected p values as indicated in the figures.

Results from alpha diversity analyses at a sequencing depth of 1498 showed *Ogg1*^*-/-*^ HFD gut microbiomes consistently yielding higher levels of diversity compared to those from the WT chow-fed animals. Chao 1 (Bonferroni-corrected *p*-values < 0.05, Fig 3A) [48, 71] and observed species analyses (Bonferroni-corrected *p*-values < 0.02, Fig 3B) revealed that the gut microbiomes from *Ogg1*^*-/-*^ (both chow and HFD) and HFD-fed WT mice were more diverse than that of chow-fed WT mice. Phylogenetic diversity analyses (tree-based; [51]) confirmed that the gut microbiomes of *Ogg1*^*-/-*^ mice, regardless of diet, were more diverse than those of WT (Bonferroni-corrected *p*-values < 0.02 for *Ogg1*^*-/-*^ vs. WT, Fig 3C). Differences observed from Shannon and Simpson indices were not statistically significant when the Bonferroni correction was applied for *Ogg1*^*-/-*^ comparisons (*p* > 0.05), however, the trend toward higher diversity in the *Ogg1*^*-/-*^ HFD gut microbiomes was observed in these tests as well (S2 Fig). Effective species transformations of these indices [72] indicated that the gut microbiomes of the HFD-fed *Ogg1*^*-/-*^ mice was approximately 1.5-fold to 2.5-fold more diverse compared to the others.

### Between sample (beta) diversity

**Fig 4:**
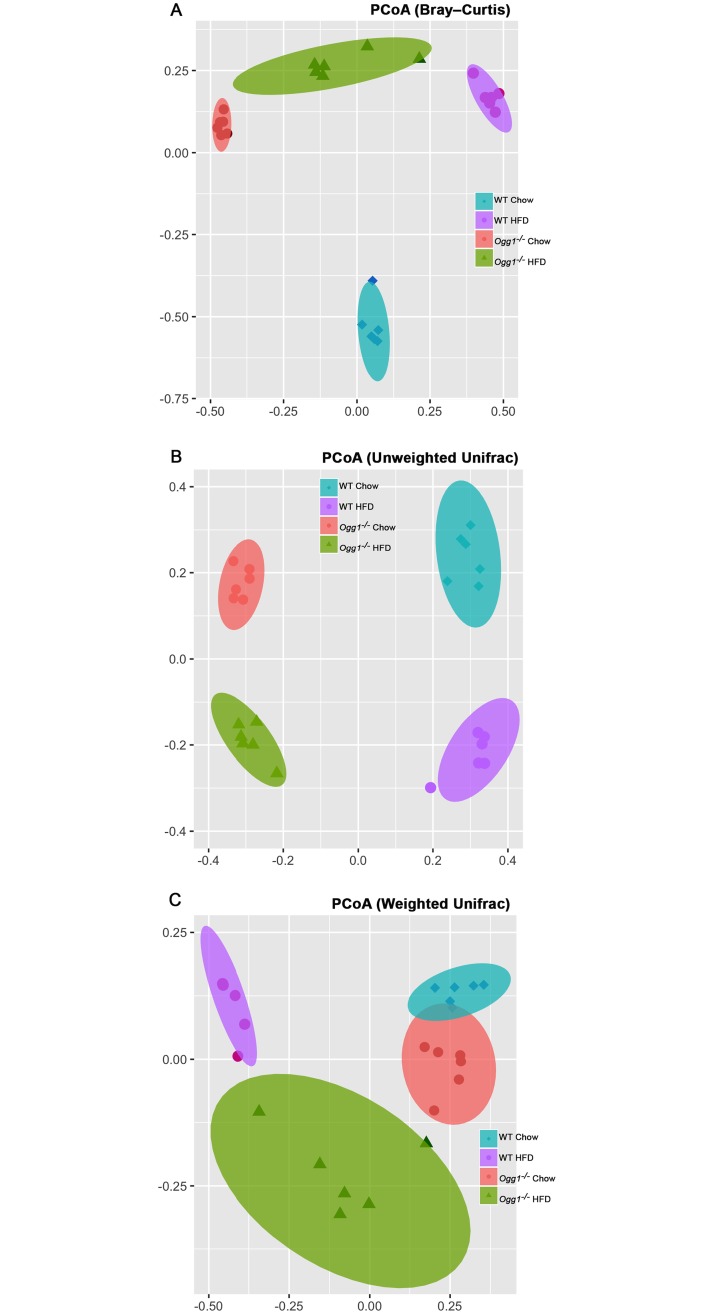
Beta diversity measures by principal coordinates analysis (PCoA). PCoA of the first 2 coordinates of the dissimilarity matrices are plotted and illustrate differences between groups, using (A) the Bray-Curtis dissimilarity matrices at an even depth of 1460 sequences; and (B) Unweighted and (C) weighted UniFrac distances using UPGMA at an even depth of 1460 sequences. 95% confidence intervals are shown by the shaded ellipses. Results are from the analysis of microbial sequences recovered from the fecal samples as described in Methods.

Clear clustering of samples by genotype and treatment was observed with principal coordinates analysis (PCoA) produced with the Bray–Curtis dissimilarity metric (Fig 4A) [54]. Nonparametric *p*-values (calculated using 999 Monte Carlo permutations) indicated significant differences in taxonomic distance in most comparisons of between-group to within-group tests (*p* < 0.027). This clustering was generally recapitulated by Unifrac PCoA using both unweighted (Fig 4B) and weighted analyses (Fig 4C), with some overlap seen in samples from WT and *Ogg1*^*-/-*^ chow-fed mice in the latter.

### Correlations

**Fig 5:**
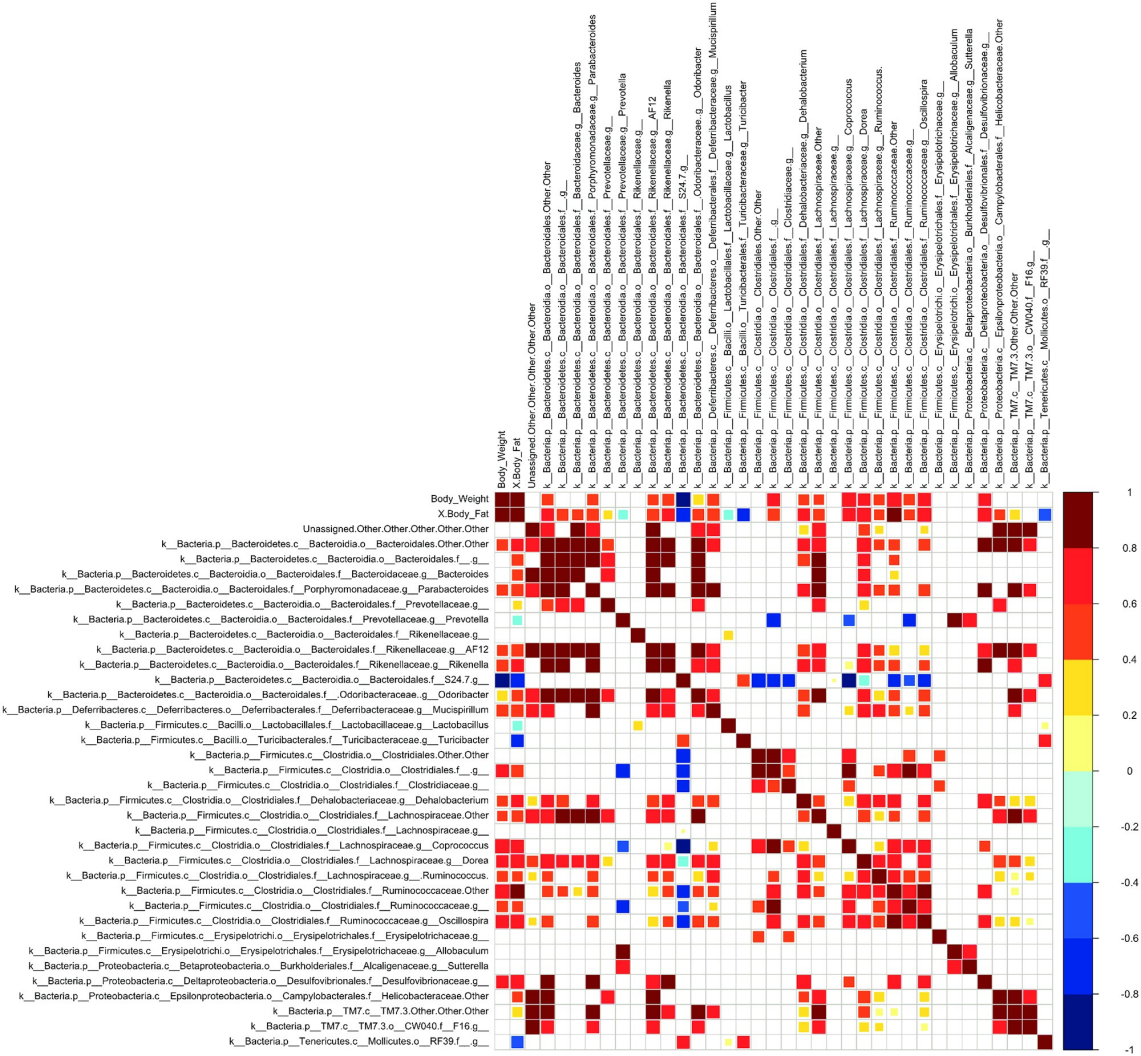
Metadata correlation heat map. Heat map shows Spearman rank correlations between host body weight, body fat, and relative abundances of different gut taxa from chow- and HFD-fed WT and *Ogg1*^*-/-*^ mice. Results are from the analysis of microbial sequences recovered from the fecal samples as described in Methods.

Spearman rank correlations between body weight, body fat and relative abundances of different taxa indicated a number of both positive and negative correlations determined to be significant (α(2) ≤ 0.01, n = 24) [73]. As anticipated, host body weight and body fat were highly correlated (ρ = 0.91). Several Firmicutes taxa were also positively correlated with body weight and body fat, including members of the genera *Coprococcus*, *Dorea*, and *Oscillospira*, in addition to several unidentified Clostridia genera (Fig 5, S1 Table). The Firmicutes genera *Dehalobacterium* and *Turicibacter* were positively and negatively correlated, respectively, with host body fat only. Several of the Bacteroidetes taxa identified, including members of the genera *Parabacteroides* and *Rikenella*, two unidentified Bacteroidia genera and Rikenellaceae strain AF12, were also significantly correlated with body fat, while *Rikenella* was additionally correlated with body weight. Bacteroidetes strain S24-7, however, was negatively correlated with both body weight and body fat. In Proteobacteria, only one Deltaproteobacterial taxon from the Desulfovibrionaceae family was positively correlated with host body weight and body fat. *Mucispirillum* (Deferribacteres phylum) was also positively correlated to body fat and body weight, while a taxon from the order RF39.f (Tenericutes phylum) was negatively correlated with both (Fig 5, S1 Table).

### Changes in gut microbiome in *Ogg1*^*-/-*^ mice are associated with increased propensity to DSS-induced colitis

**Fig 6:**
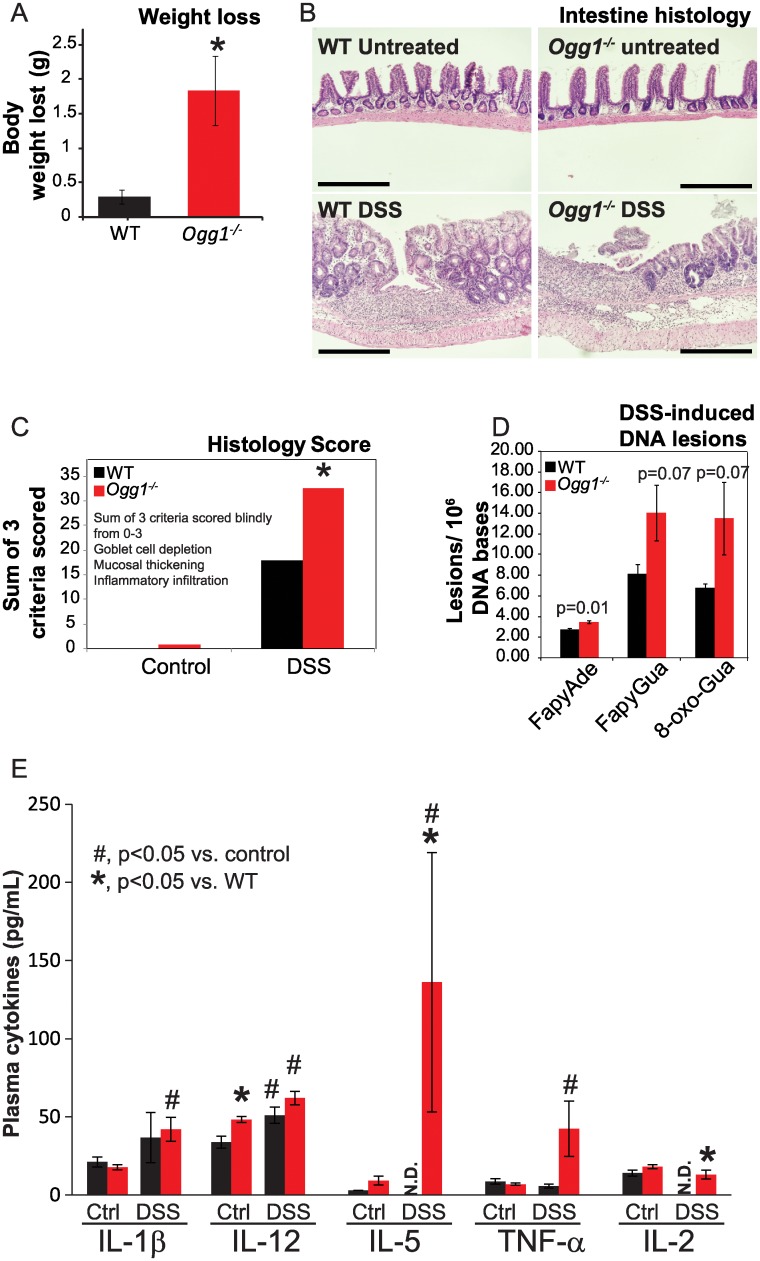
DSS-induced inflammation and colitis in *Ogg1*^*-/-*^ mice. (A) Weight loss was measured after 5 days of oral DSS administration; (B) Intestinal structure was visualized by H&E staining of formalin-fixed paraffin-embedded sections; scale bars represent 220 μm; (C) Intestine sections were assigned a histological score by blinded scoring of 3 criteria, including goblet cell depletion, mucosal thickening, and inflammatory infiltrate; (D) Oxidative DNA lesions were quantified by GC-MS/MS; (E) Serum levels of inflammatory cytokines were determined by Luminex multiplexed immunoassay.

In analyzing the marked alterations in the gut microbial profile of *Ogg1*^*-/-*^ mice, we were struck by the observation that several of the changes in gut microbes in *Ogg1*^*-/-*^ mice have been reported to play a role in intestinal inflammation and colitis. For instance, increased levels of Deferribacteres, *Mucispirillum*; and Proteobacteria, particularly *Sutterella* have been shown to be associated with active colitis [74]. Similarly, an expansion of TM7 has been associated with inflammation and risk for colitis [75]. In light of these coordinated increases in bacterial species associated with inflammation in both chow-fed and HFD-fed *Ogg1*^*-/-*^ mice, we asked the question of whether *Ogg1*^*-/-*^ mice would have increased sensitivity to an acute inflammatory challenge. To induce an acute inflammatory condition in the colon, WT and *Ogg1*^*-/-*^ mice were challenged with 3% DSS in 5% sucrose in water for 5 days. Mice were euthanized either at the end of the exposure or after being transferred to regular water for 5 additional days. In contrast to wild-type controls, by the end of the 10 day protocol, *Ogg1*^*-/-*^ mice experienced considerably greater weight loss (Fig 6A) and lethargy, and had severe diarrhea with evidence of blood in the fecal matter. Colonic tissues were harvested for ultrastructural examination, and epithelial cells harvested for DNA damage assessment (Fig 6B). Histopathologic analyses for induction of acute ulcerative colitis were based on goblet cell depletion, mucosal thickening, and inflammatory infiltrate (Fig 6C). These data revealed that control (no DSS treatment) tissues were unremarkable for WT mice, while the colons of OGG1-deficient mice showed mild symptoms of pre-existing disease (Fig 6B and 6C). Following the 10 day protocol, wild-type tissues showed moderate ulcerative colitis. In contrast, tissues derived from *Ogg1*^-/-^ mice were twice as severely affected (~2-fold), confirming a much more severe inflammatory response in the *Ogg1*-deficient mice (Fig 6B). Analyses by GC-MS/MS of the levels of DNA base lesions revealed trends for increased levels of three lesions, namely 4,6-diamino-5-formamidopyrimidine (FapyAde), FapyGua, and 8-oxo-Gua in OGG1 deficient-mice (Fig 6D), correlating with colonic inflammation in these animals.

### *Ogg1*^*-/-*^ mice have elevated plasma levels of pro-inflammatory cytokines

To determine if the elevated markers of intestinal inflammation were accompanied by increased plasma markers of inflammation, we measured levels of several plasma pro-inflammatory cytokines in WT and *Ogg1*^-/-^ mice before and after 5 days of DSS treatment. Levels of the inflammatory cytokine interleukin (IL)-1β levels were upregulated by DSS treatment in WT and *Ogg1*^*-/-*^ mice, but this difference reached statistical significance only in the latter genotype (Fig 6E). IL-12 levels were increased in *Ogg1*^*-/-*^ mice even under baseline conditions and were further increased by DSS treatment in both genotypes (Fig 6E). Levels of IL-5, secreted by T-helper cells and mast cells and involved in activation of eosinophils [76], were significantly elevated in DSS-treated *Ogg1*^*-/-*^ mice but were undetectable in WT animals (Fig 6E). Levels of the pro-inflammatory cytokine TNFα were elevated by over 7-fold on average in DSS-treated *Ogg1*^*-/-*^ mice but did not reach statistical significance due to inherent variability in plasma TNFα levels (Fig 6E). IL-12 is a macrophage-derived cytokine that induces T-helper (Th) cell polarization and Th1 T-cell differentiation and is elevated in intestinal inflammatory disease [77]. IL-12 levels were increased by DSS-treatment in both genotypes. However, interestingly, baseline levels of IL-12 were significantly higher in *Ogg1*^*-/-*^ mice (Fig 6E), suggesting the presence of a pro-inflammatory baseline state in these mice that may pre-dispose them to both metabolic and inflammatory diseases.

## Discussion

The intestinal microbiome plays a critical role in altering whole body energy homeostasis, and several studies have focused on the role of dietary components or pathological states in eliciting alterations in the gut microbiome [31, 38, 78–80]. By virtue of its high rate of turnover, intestinal cells are particularly vulnerable to the effects of persistent DNA damage. However, to our knowledge, there are no studies examining the role of oxidatively-induced DNA damage or host repair capacity influencing the gut microbiome. To understand if the metabolic abnormalities observed in *Ogg1*^-/-^ mice are related to alterations in the gut microbial population, microbial diversity and identity were determined in this study.

One of the most striking findings from these studies was the significantly increased alpha diversity in fecal samples from *Ogg1*^*-/-*^ mice (Table 1, Fig 3). While the *Firmicutes* (F) and *Bacteroidetes* (B) phyla represented nearly 100% of microbial diversity in feces from chow-fed WT mice, these phyla only contributed to 88% of the bacterial population in chow-fed *Ogg1*^*-/-*^ mice. Contributing to this lower overall *F+B* % in the *Ogg1*^*-/-*^ group, there was a statistically significant decrease in the *Firmicutes* (p = 0.036), and a significant reduction in the *F*:*B* ratio in *Ogg1*^*-/-*^ mice (0.30 in WT-chow vs. 0.20 in *Ogg1*^*-/-*^ chow). Thus, although reduced *F*:*B* ratios have been associated with improved metabolic phenotypes in some reports [31, 78, 79], this correlation was not observed in our model. Indeed, other studies have recently indicated that this ratio is not always predictive of host pathology and that the relationships between the gut microbiota and host physiology extend beyond these two phyla [81–83]. Interestingly, 12–21% of the bacteria observed in *Ogg1*^*-/-*^ mice were in phyla (*Proteobacteria*, TM7, Deferribacteres, and an unassigned phylym) unique to this genotype (Fig 2, Table 1). At the genus level, the most remarkable differences were in the abundance of *Prevotella*, in the family *Prevotellaceae* (Family: *Bacteroidetes*). This bacterial group, found only in the *Ogg1*^*-/-*^ animals, is known to degrade cellulose and xylans and has been associated with increased energy harvest and fecal short-chain fatty acids (SCFAs) [84], providing a potential mechanistic link between the increased body weights and adiposity of *Ogg1*^*-/-*^ mice and their altered microbiomes.

With regard to colonic inflammation and associated changes in the gut microbiome, several notable alterations were observed in *Ogg1*^*-/-*^ mice. For instance, we observed significant increases in the relative abundance of Prevotellacaea and TM7 groups. Elinav *et al*. [75] reported similar increases in these bacteria, together with a coincident decrease in the proportion of *Lactobacillus* in *Nlrp6*^*-/-*^ mice, in another model where modulation of the host genotype alone resulted in dramatic alterations in the intestinal microbial environment. NLRP6 is a sensor of both endogenous and exogenous stress and is involved in the production of IL-18, which is critical for the maintenance of the intestinal epithelial cell barrier. *NLRP6*^*-/-*^ mice exhibit reduced IL-18 levels in intestinal epithelial cells and, interestingly, like *Ogg1*^*-/-*^ mice were highly susceptible to DSS-induced colitis. The correlation between colonic inflammation and changes in pro-inflammatory taxa such as Prevotella and TM7 in the *Ogg1*^*-/-*^ intestines suggests that local changes in the intestine may mediate this pro-inflammatory phenotype.

In addition, a number of other taxa in the Bacteriodetes phylum (*Allobaculum*, *Rikenella*, *Bacteroides*, *Parabacteriodes*, *Odoribacteria*, and an unidentified group of the order Bacteriodales) were also present in significant numbers only in *Ogg1*^*-/-*^ gut microbiomes. This was also true for the Firmicutes genus *Dorea*, Proteobacteria taxa Sutterella, and Helicobacteraceae (other), Deferribacteres (Mucillispirillum) and an unassigned taxon. In addition to being positively correlated with body weight and body fat in this study, increases in *Dorea* have been correlated with the onset of nonalcoholic fatty liver and its progression to nonalcoholic steatohepatitis in pediatric patients [85]. Dorea was also part of a microbial cohort uncovered using a Random Forest-trained model to predict disease state in a study on relapsing remitting multiple sclerosis (Chen, 2016). In further support of the bacterial changes in *Ogg1*^*-/-*^ mice being associated with increased inflammatory status, *Mucispirillum*, an intestinal commensal normally associated with the gut epithelium and found to be increased in *Ogg1*^*-/-*^ animals, has been shown to activate T cell–dependent immunoglobulin A (IgA) production and to increase in abundance in the presence of inflammation [86]. In addition, while Bacteroidetes Rikenellaceae AF12 taxa were also found to be present in WT gut microbiomes (but < 0.01% compared to *Ogg1*^*-/-*^ gut microbiomes and > 5% in *Ogg1*^*-/-*^ HFD), these microorganisms are of note as they have been reported to increase in the gut microbiomes of mouse models exhibiting DSS-induced or spontaneous chronic colitis in comparison to wild-type controls [87]. Finally, Proteobacteria in the Helicobacteriace and Sutterella groups have been reported to be elevated under conditions of DSS-induced colitis [74, 88]. It is notable that many of these pro-inflammatory changes were present in chow-fed *Ogg1*^*-/-*^ mice and were further exacerbated in HFD-fed animals. This baseline increase in inflammatory species is consistent with our observations of increased underlying inflammatory histological changes in *Ogg1*^*-/-*^ mice, even prior to DSS administration (Fig 6B and 6C). Our findings of increased inflammation in *Ogg1*^*-/-*^ intestines are also consistent with previous demonstrations of increased neuronal inflammation in OGG1-deficient mice [23].

Interestingly, our observations of increased intestinal inflammation in *Ogg1*^*-/-*^ mice are divergent from reports of reduced inflammatory responses in these mice, when exposed to inhaled challenges. Investigations of pulmonary inflammation in *Ogg1*^*-/-*^ and knocked down mice have been reported using ovalbumin or ragweed pollen extract as pro-inflammatory challenges [89–91]. In these studies, deficiency in OGG1 correlated with significantly less severe responses, including less extensive inflammatory cell infiltration, reduced oxidative damage and decreased activation of Th2-associate genes including STAT6, TNFα, INFγ, IL2, 4, 5, 6,10,13, and 17 relative to wild-type animals [89–91]. *Ogg1*^*-/-*^ mice were also significantly protected from the inflammatory responses to *Helicobacter pylori* infection [92]. Further, a recent study indicated that pharmacological inhibition of OGG1 resulted in decreased OGG1 binding to guanine-rich promoters of pro-inflammatory genes, thereby reducing pro-inflammatory gene expression in mouse lungs [93]. These data, combined with our observations of increased inflammation in intestines of *Ogg1*^*-/-*^ mice, point to divergent tissue-specific effects of OGG1 deficiency with regard to inflammation.

Similar to *Ogg1*^*-/-*^ mice, mice that were knocked out for the DNA repair proteins alkyladenine DNA glycosylase (*Aag)*, AlkB homology (*Alkbh)2* or *Alkbh3* were all greatly sensitized to the development of severe ulcerative colitis, following DSS treatments [94, 95]. *Aag*^*-/-*^ mice that were challenged with an acute DSS regimen accumulated significantly greater quantities of DNA base damage caused by reactive oxygen and nitrogen species, and showed severe colonic damage [94]. In addition to increased sensitivity of the single knockouts, a single DSS treatment proved lethal to triple *Aag*, *Alkbh2*, and *Alkbh3* knockout animals, thus demonstrating the essential role of the initiation of BER in colonic inflammation. This investigation also demonstrated that shifting from an acute to a chronic DSS challenge coupled with a single dose of azoxymethane resulted in a significantly increased colonic tumor burden [94]. Increased tumor burden has also been demonstrated in *Ogg1*^*-/-*^ mice that were exposed to conditions of chronic DSS treatment [96]. This study also showed evidence of increased ulcerative colitis, thus suggesting that deficiencies in OGG1 can have either protective or sensitizing effects on disease outcomes depending on the organ system under investigation. Interestingly, knockout of the MUTYH DNA glycosylase involved in excision of mispaired adenines across from 8-oxo-Gua, was previously shown to be protective against the effects of a single-cycle or chronic DSS administration, despite greater induction of 8-oxo-Gua lesions in these mice [97]. These results are puzzling in light of the fact that MUTYH deficiency predisposes animals to spontaneous and KBrO3 induced intestinal carcinogenesis and humans and mice to colorectal cancers [98–102]. The lack of a colitis response in *Mutyh*^*-/-*^ mice was attributed to a role for MUTYH in mounting an inflammatory response [97]. Any potential modulation of the intestinal microbiome, or of responses to hypercaloric diets, has not been investigated in these models of DNA repair defects.

While the best described role for OGG1 is in excision of oxidized guanine bases through the base-excision DNA repair pathway, it should be noted that additional functions for OGG1 have been described. For instance, binding of OGG1 8-oxoG upstream in promoter regions of pro-inflammatory genes has been shown to upregulate their expression in lung cells [93, 103]. Therefore, it is possible that non-canonical functions of OGG1 may mediate the pro-inflammatory responses seen in intestines of *Ogg1*^*-/-*^ mice. If so, our data would again suggest that transcriptional regulation by OGG1 occurs in a tissue-specific manner, with OGG1 binding promoting inflammation in the enterocyte while being anti-inflammatory in the lung. Alternatively, it is plausible that upon acute induction of severe DNA damage across the genome, as might occur during acute colitis, channeling and sequestration of OGG1 to its DNA repair functionality may result in its reduced availability as a transcriptional regulator.

Correlating with the observed changes in tissue histology and intestinal pro-inflammatory microbes, we observed several significant alterations in inflammatory cytokines in DSS-treated WT and *Ogg1*^*-/-*^ mice. For instance, our observation of significantly elevated IL-1β (Fig 6E) in *Ogg1*^*-/-*^ mice is consistent with the inflammation observed by tissue histology in these animals (Fig 6B and 6C). IL-1β is a potent inflammatory cytokine that is implicated in the development of colitis and inflammatory responses in the gut [104, 105]. Furthermore, very high levels of IL-1β have been reported in the intestines of patients suffering from inflammatory bowel diseases [106–109]. Intriguingly, levels of another interleukin, IL-12, were elevated under baseline conditions in *Ogg1*^*-/-*^ mice [106–109], suggesting the presence of increased basal inflammation in these mice that may contribute to increased susceptibility to colonic inflammation. Notably, antibodies that neutralize IL-12 have been shown to suppress chronic intestinal inflammation in mice [77]. IL-5 levels, while undetectable in WT animals, were markedly elevated in *Ogg1*^*-/-*^ mice upon DSS treatment (Fig 6E). IL-5 stimulates production and activation of eosinophils and also enhances the production of immunoglobulin A (IgA). However, while tissue eosinophilia is commonly observed in inflammatory bowel disease and colon inflammation, the role of IL-5 in mediating intestinal pathology is not clear.[76] A recent study indicated that tissue eosinophils may serve to protect against tissue injury in DSS-induced colitis.[110] Our observation of a remarkable increase in serum IL-5 levels in *Ogg1*^*-/-*^ mice (Fig 6E) may thus correspond with the severity of inflammation in *Ogg1*^*-/-*^ animals and may signal a compensatory response to tissue injury in these mice.

Taken together, our results reveal a novel role for host DNA repair status and OGG1 genotype in modulating intestinal inflammation and the intestinal microbiome. At present, we do not fully understand how DNA repair deficits in the epithelium elicit changes in inflammation and the intestinal microbiome. Since alterations in both the microbiome composition, as well as underlying intestinal inflammation were observed in young chow-fed mice prior to challenge with metabolic or inflammatory challenges such as HFD or DSS, it is not clear whether our observed alterations in DNA damage precede or follow the observed changes in the intestinal microbiome. However, these data demonstrate that OGG1 deficiency sensitizes mice to obesity and intestinal inflammation along with marked alterations in the intestinal microbiome that support enhanced energy harvest and inflammation.

## Supporting information

S1 Table Spearman rank correlation matrix of gut microbial sequences recovered from chow- and HFD-fed WT and *Ogg1*^*-/-*^ mice. Results are from the analysis of sequences recovered from the fecal samples as described in Methods.
(XLSX)

S1 Fig Goods coverage. Rarefaction curves generated from the Goods coverage of OTUs (index > 0.99) across samples.
(EPS)

S2 Fig Shannon (H´) [52] and Simpson (B) diversity indices. Results are from the analysis of sequences recovered from the fecal samples as described in Methods.
(EPS)
